# Targeted Activation of Cholinergic Interneurons Accounts for the Modulation of Dopamine by Striatal Nicotinic Receptors

**DOI:** 10.1523/ENEURO.0397-17.2018

**Published:** 2018-10-30

**Authors:** Katherine R. Brimblecombe, Sarah Threlfell, Daniel Dautan, Polina Kosillo, Juan Mena-Segovia, Stephanie J. Cragg

**Affiliations:** 1 Department of Physiology, Anatomy and Genetics, Sherrington Building, Oxford, UK; 2 Oxford Parkinson’s Disease Centre, University of Oxford, Oxford OX1 3PT, UK; 3 MRC Anatomical Neuropharmacology Unit, Department of Pharmacology, Oxford, UK; 4 Center for Molecular and Behavioral Neuroscience, Rutgers University, Newark, NJ

**Keywords:** Acetylcholine, brainstem, cholinergic interneurons, dopamine, nicotinic receptors, striatum

## Abstract

Striatal dopamine (DA) is a major player in action selection and reinforcement. DA release is under strong local control by striatal ACh acting at axonal nicotinic ACh receptors (nAChRs) on DA axons. Striatal nAChRs have been shown to control how DA is released in response to ascending activity from DA neurons, and they also directly drive DA release following synchronized activity in a small local cholinergic network. The source of striatal ACh has been thought to arise solely from intrinsic cholinergic interneurons (ChIs), but recent findings have identified a source of cholinergic inputs to striatum from brainstem nuclei, the pedunculopontine nucleus (PPN) and laterodorsal tegmentum (LDT). Here, we used targeted optogenetic activation alongside DA detection with fast-scan cyclic voltammetry to test whether ChIs alone and/or brainstem afferents to the striatum can account for how ACh drives and modulates DA release in rat striatum. We demonstrate that targeted transient light activation of rat striatal ChIs drives striatal DA release, corroborating and extending previous observations in mouse to rat. However, the same light stimulation targeted to cholinergic brainstem afferents did not drive DA release, and nor did it modulate DA release activated subsequently by electrical stimulation, whereas targeted activation of ChIs did so. We were unable to obtain any evidence for DA modulation by PPN/LDT stimulation. By contrast, we could readily identify that striatal ChIs alone are sufficient to provide a source of ACh that powerfully regulates DA via nAChRs.

## Visual Abstract

**Figure:**
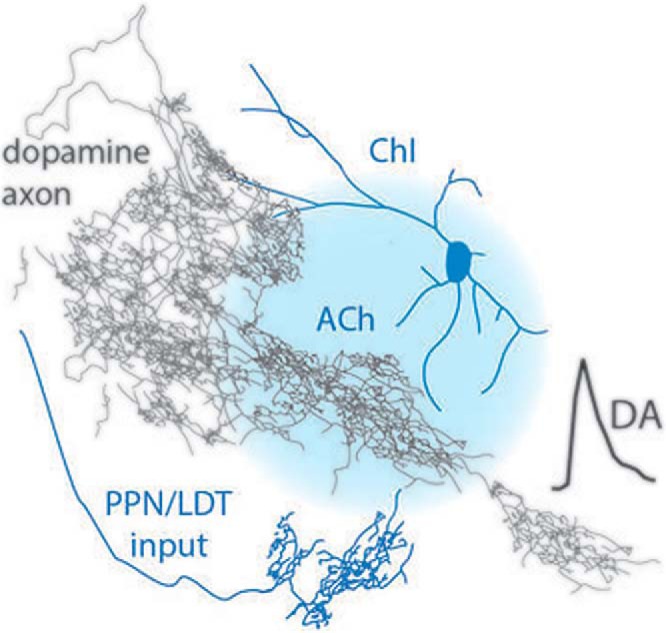

## Significance Statement

Striatal acetylcholine (ACh) can powerfully regulate striatal dopamine (DA). Until recently, cholinergic interneurons were thought to be the only source of striatal ACh, but brainstem cholinergic neurons have now been revealed to innervate striatum. Here, we use targeted optogenetic activation in rats to explore which input accounts for the rapid regulation of striatal DA release. We find that targeted activation of striatal cholinergic interneurons in rat striatal slices can reproduce how striatal ACh drives and modulates dopamine release in mouse, but we could not find comparable evidence for a role for brainstem cholinergic inputs. Activation of cholinergic interneurons alone can reproduce the previously documented effects of activating all striatal ACh systems on DA release.

## Introduction

The striatum is the primary input-receiving nucleus of the basal ganglia, and the modulation of information flow through the striatum is crucial to basal ganglia function including motor control and reward-driven behaviors. Two key striatal neuromodulators are dopamine (DA) and acetylcholine (ACh). Striatal ACh plays powerful roles not only in regulating excitability of striatal neurons, but also in directly regulating DA transmission (Zhou et al., 2001; Rice and Cragg, 2004; Zhang and Sulzer, 2004; Cragg, 2006; Threlfell et al., 2010, 2012; Cachope et al., 2012). Activation of β2-containing nAChRs on dopaminergic axons (Jones et al., 2001) promotes DA release probability, but in turn subsequently leads to short-term depression of further release, making DA release insensitive to depolarization frequency (Rice and Cragg, 2004; Zhang and Sulzer, 2004; Wang et al., 2014). It has therefore been proposed that *in vivo*, when striatal cholinergic interneurons (ChIs) pause their firing in response to salient and conditioned stimuli (Aosaki et al., 1994; Apicella et al., 1997), or when nicotine causes receptor desensitization, nAChRs will turn off and allow DA release to better reflect the frequency of activity in DA neurons (Rice and Cragg, 2004; Cragg, 2006). In addition, synchronized activation of a small network of striatal cholinergic inputs can directly drive DA release via activation of striatal nAChRs, bypassing activation of DA neurons in the midbrain (Cachope et al., 2012; Threlfell et al., 2012).

The source of striatal ACh has long been thought to be exclusively striatal ChIs, which constitute only ∼1%–2% of all striatal neurons (Kawaguchi et al., 1995; Rymar et al., 2004). Despite being few in number, ChIs have an extensive influence throughout the striatum through their extended dendritic and axonal arbors (Hoover et al., 1978; Graveland and DiFiglia, 1985; Kawaguchi, 1993; Descarries and Mechawar, 2000). However, recent work has revealed an additional source of ACh inputs to striatum in rats that arise from the pedunculopontine nucleus (PPN) and laterodorsal tegmental nucleus (LDT) of the brainstem. These inputs project respectively to CPu and NAc (plus medial CPu), express the vesicular ACh transporter (vAChT), and give rise to striatal synapses (Dautan et al., 2014, 2018). The identification of this brainstem input to striatum has led to the question of whether these inputs provide a source of ACh that might mediate the previously documented effects of ACh on striatal DA.

Previous studies of DA regulation using strategies that target optogenetic constructs to striatal ChIs (Cachope et al., 2012; Threlfell et al., 2012) have primarily used an AAV serotype (AAV5) that does not select for anterograde over retrograde expression, and thus intrinsic striatal ChIs as well as any ACh afferent inputs will have expressed channelrhodopsin2 (ChR2) in those studies. Therefore, we explored here the roles of cholinergic brainstem afferents versus ChIs as sources of the striatal ACh that drives and modulates striatal DA release (Zhou et al., 2001; Rice and Cragg, 2004; Exley et al., 2008, 2012; Zhang et al., 2009; Threlfell et al., 2010, 2012; Cachope et al., 2012), by targeting expression of ChR2 to striatal ChIs or to cholinergic brainstem neurons. We used rats as a species of choice because brainstem cholinergic input has been best described in this species (Dautan et al., 2014) and also, to validate the role of ACh in DA regulation across rodent species.

## Methods

### Stereotaxic surgery

Adult (250 g - 350 g) male Long Evans ChAT::cre^+^ rats were maintained on a 12-h light/dark cycle (light on at 7:00 am) and had *ad libitum* access to water and food. All procedures were performed in accordance with the Society of Neuroscience policy on the use of animals in neuroscience and the Animals (Scientific Procedures) Act, 1986 (UK), under the authority of a Project License approved by the Home Office and the local ethics review committee.

All surgical procedures were performed during deep isoflurane anesthesia (2% in O_2_, IsoFlo, Schering-Plow). ChAT::cre^+^ rats were injected in each hemisphere with adeno-associated virus (AAV) of serotype 2 for anterograde-specific expression (Salegio et al., 2013; Dautan et al., 2016), carrying the fusion genes for the enhanced yellow fluorescent protein (eYFP) and channelrhodopsin (ChR2; AAV2-EF1a-DIO-ChR2-eYFP, Gene Therapy Center Virus Vector Core, University of North Carolina). The viral vectors were injected in the right brainstem (3 injections of 500 nl each in the LDT: AP –8.5, ML +1.0, DV –6.0, the caudal part of the PPN: AP –7.8, ML +1.8, DV –6.8, and the rostral part of the PPN: AP –7.3, ML +1.8, DV 7.2) and in the left striatum to target ChIs (4 injections of 500 nl each in the ventral DLS: AP +0.5, ML +3.0, DV –5.0; dorsal DLS: AP +0.5, ML +2.0, DV –4.0; ventral DMS: AP +0.5, ML +2.0, DV –5.0; dorsal DMS: AP +0.5, ML +3.0, DV –4.0). All injections were made using a 1-µl syringe (Neuros 7001, Hamilton) at a rate of 50 nl/min and left to diffuse for 5 min before retraction of the syringe. Animals were monitored during recovery. LDT/PPN injections result in ChR2-eYFP expression in 66% ± 7% of LDT/PPN cholinergic neurons, with 91.4% ± 0.5% of ChR2-eYFP neurons being immunopositive for ChAT. Light activation of these inputs can successfully drive a cholinergic output from terminals in VTA (Dautan et al., 2016).

### Slice preparation

Six to eight weeks after AAV2 injections, rats were killed by decapitation under isoflurane-induced anesthesia, and brains were rapidly removed; 300-µm coronal striatal sections were taken in ice-cold buffer containing, in mm: 120 NaCl, 20 NaHCO_3_, 6.7 HEPES acid, 5 KCl, 3.3 HEPES salt, 2 CaCl_2_, 2 MgSO_4_, 1.2 KH_2_PO_4_, and 10 glucose, saturated with 95% O_2_/5% CO_2_. Slices were kept at room temperature in HEPES-based buffer for at least 1 h.

### Fast-scan cyclic voltammetry (FCV)

DA release was monitored in acute slices using FCV. Slices were superfused in a recording chamber with bicarbonate-buffered artificial cerebrospinal fluid (aCSF) containing, in mm: 124.3 NaCl, 26 NaHCO_3_, 3.8 KCl, 2.4 CaCl_2_, 1.3 MgSO_4_, 1.23 KH_2_PO_4_, and 10 glucose, saturated with 95% O_2_/5% CO_2_ at 31–33°C. Evoked extracellular DA concentration ([DA]_o_) was monitored using FCV at 7–10-µm-diameter carbon-fiber microelectrodes (CFM) fabricated in-house (tip length 50–100 µm) and a Millar voltammeter (Julian Millar, Barts and the London School of Medicine and Dentistry). In brief, a triangular voltage wave form (range –700 to +1300 mV versus Ag/AgCl) was applied at 800 V/s at a scan frequency of 8 Hz. Electrodes were switched out of circuit between scans. Electrodes were calibrated using 1–2 μm DA in each experimental medium. Calibration solutions were prepared immediately before calibration from a 2.5-mm stock solution in 0.1 m HClO_4_ stored at 4°C. Signals were attributable to DA by the potentials for peak oxidation and reduction currents (oxidation peak: +500–600 mV, reduction peak: ∼–200 mV).

### Electrical stimulation

DA release was evoked by a local bipolar concentric Pt/Ir electrode (25-µm diameter; FHC) placed ∼100 µm from the CFM. Stimulus pulses (200-µs duration) were given at 0.6 mA (perimaximal in control conditions). Electrical stimulations were repeated at 2.5-min intervals, which allow stable release to be sustained over several hours. Each stimulus type was repeated in triplicate in a random order. When directly comparing DA release evoked by light versus electrical stimuli, stimuli at 25 Hz were used to allow for ChR2 reactivation. When exploring changes in frequency sensitivity of DA release, electrical stimulations of single pulses (1p) and 4 pulses (4p) at 100 Hz were used because the ratio of DA released by 4p/100Hz versus 1p (4p:1p) is very sensitive to nAChR activity (Rice and Cragg, 2004). When nAChRs are active, 4p:1p is ∼1, indicating a large degree of short-term depression, whereas when nAChRs are blocked or desensitized, the 4p:1p can be ∼4.

### Optical stimulation

Light stimulation of ChR2-expressing ChIs and brainstem afferents in striatum was via a 470-nm LED (OptoLED, Cairn Research), which illuminated the full field of view (2.2-mm diameter, 10× water-immersion objective). TTL-driven light pulses (2-ms duration, ∼6.5 mW, Thor labs optical power meter) were applied singly or in trains (4–10 pulses, 10–25 Hz). In some experiments, light activation of ChIs that was subthreshold for evoking DA release was desired, for comparison with brainstem activation. This was achieved by stimulating and recording in areas of sparse ChI transfection, i.e., posterior and lateral CPu. Electrical and optical stimulations at a given site were alternated.

### Drugs

Dihydro-β-erythroidine (DHβE) was purchased from Ascent Scientific. All other reagents were purchased from Sigma Aldrich. Stock solutions were made to 1000× final concentrations in H_2_O and stored at –20°C. Drugs were diluted to their required concentrations in aCSF immediately before use.

### Immunocytochemistry and site marking

Images of ChR2-eYFP expression were captured after fixation. Slices were fixed (PFA 4% formaldehyde) for at least 2 d, washed in PBS, and resectioned to 40 μm. Sections were mounted on gelled slides with Vectashield (Vector labs), and ChR2-eYFP fluorescence was imaged using an Olympus BX41 microscope with Q-Click cooled monochrome CCD camera (Olympus Medical).

ChR2-eYFP fluorescence in ChIs was readily visible in 300 µm acute living brain slices in the recording chamber under water-immersion optics using (nonpulsing) activation of eYFP using OptoLED (505 nm), whereas ChR2-eYFP-expressing fibers from PPN/LDT were not. We therefore marked striatal recording sites with FluoSpheres, to determine *post hoc* whether they were within an area of ChR2-expressing cholinergic brainstem innervation. Recording sites were located in central to medial striatum consistent with the organization of cholinergic inputs to striatum from PPN/LDT and were labeled by replacing the CFM with a micropipette and injecting 0.5-1.0 μl of 1-μm-diameter red FluoSpheres (Invitrogen) after recordings. Slices were fixed (PFA 4% formaldehyde) for at least 2 d, washed in PBS, and resectioned to 40 μm. Sections were mounted on gelled slides with Vectashield (Vector labs) and imaged using an Olympus BX41 microscope with Q-Click cooled monochrome CCD camera (Olympus Medical). Monochrome images of striatal ChR2-eYFP expression and FluoSpheres were each captured and pseudo-colored using Q-capture Pro7 and optimized using histogram equalization. Only recordings that were confirmed *post hoc* to be in striatal areas within a region of ChR2-eYFP–expressing neuropil (see example in Fig. 2A) were included for analysis.

To confirm that striatal neurons transduced by AAV2 injected in rat striatum were cholinergic, striatal sections were incubated in a blocking solution consisting of 10% normal donkey serum (NDS) in PBS containing 1% Triton X-100 for 1 h minimum. Sections were incubated with previously validated antibodies against GFP (1:1000, raised in rabbit; Invitrogen, A21311) and an antibody against ChAT (raised in goat; 1:500 dilution in 1% NDS, 0.03% Triton X-100 in PBS; Millipore, AB144P) followed by several washes in PBS and incubation in an Alexa Fluor 488–conjugated donkey anti-rabbit antibody (1:1000 dilution, Jackson Immunoresearch) or CY5-conjugated donkey anti-goat antibody (1:1000, Jackson Immunoresearch).

### Statistics

Data are expressed as mean ± SEM. Data at each site were averaged from at least three repeat recordings for each stimulus, and where appropriate was normalized to 1p release in control conditions. Population means were compared using two-way ANOVA with repeated measures and Sidak’s multiple comparison *t* test as appropriate. Superscript letters listed with *p*-values correspond to the statistical tests shown in Table 1.

**Table 1.** Statistical analysis

Location	Data structure	Type of test	Power
a	Normal	2-way ANOVA with Sidak posttest	Interaction: *F*_1,11_ = 8.5, *p* = 0.014; *post hoc* test: 1p vs 4p DHβE, *p* < 0.05
b	Normal	2-way ANOVA	Interaction: *F*_1,6_ = 17.1, *p* = 0.006; *post hoc* test: 1p vs 4p, *p* < 0.05
c	Normal	2-way ANOVA (repeated measures)	Effect of light: *F*_1,4_ = 0.19, *p* = 0.68; pre-flash × pulse number interaction, *F*_1,4_ = 0.02, *p* = 0.88
d	Normal	2-way ANOVA	Effect of region: *F*_1,18_ = 8.7, *p* = 0.004; posttest, with vs without prepulse: ChIs, *p* < 0.01
e	Normal	2-way ANOVA	Effect of light: *F*_1,12_ = 0.2, *p* = 0.67

## Results

### Striatal ACh from rat ChIs drives dopamine release

**Figure 1.**
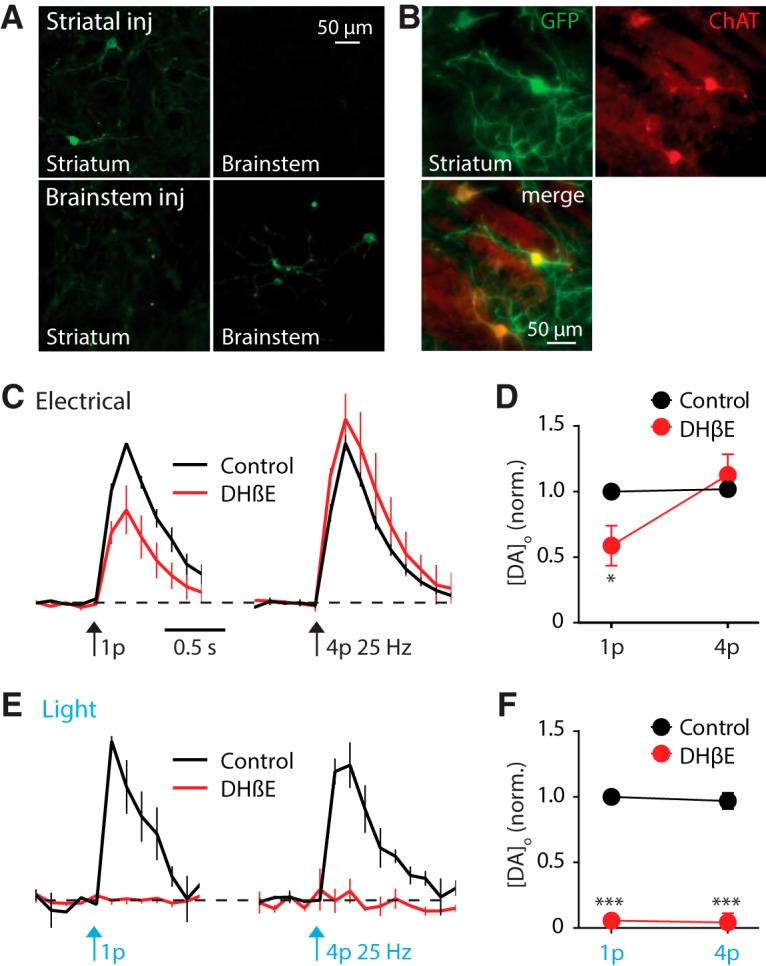
Light activation of rat striatal cholinergic interneurons evokes striatal dopamine release. ***A***, ChR2-eYFP detected in striatum (left) and PPN/LDT (right) after AAV2 injection to PPN/LDT (lower), and detected in striatum only after injection to striatum (upper), fixed tissue. ***B***, GFP-immunopositive soma after striatal injection were co-immunopositive for ChAT. ***C***, ***E***, Mean profiles of [DA]_o_ ± SEM versus time evoked by 1 or 4 pulses (25 Hz) of electrical (C) or light (E) stimulation, in control drug-free conditions or in the presence of DHβE (1 μm). Data are normalized to 1p peak [DA]_o_ in control conditions. Control conditions (black lines), DHβE (red lines). ***D***, ***F***, Mean peak [DA]_o_ normalized to control 1p for electrical (***D***) and light-evoked DA release (***F***) in control (black) and in the presence of DHβE (red). *n* = 3 rats, *n* = 4 sites. Two-way ANOVA with Sidak posttest comparisons of control versus DHβE: *p < 0.05, ***p < 0.001.

To obtain striatal expression of ChR2-eYFP in either ChIs or afferents from PPN/LDT, we injected anterograde-specific AAV2-packaged constructs into either the striatum or PPN/LDT, respectively, of ChAT-Cre rats. Striatal injections resulted in detectable ChR2-eYFP expression in striatum and an absence of retrograde transduction of ChR2-eYFP expression in PPN/LDT cholinergic neurons in brainstem, whereas PPN/LDT injections resulted in ChR2-eYFP expression in PPN/LDT and in neuropil in striatum (Fig. 1*A*), as shown previously following striatal versus PPN/LDT injections of AAV2-EF1a-DIO-YFP in ChAT-Cre rats (Dautan et al., 2014). ChR2-eYFP expression was confirmed as originating from neurons immunopositive for ChAT in striatum (Fig. 1*B*), as has been confirmed previously for expression in PPN/LDT neurons following brainstem injections (Dautan et al., 2016).

We first sought to test whether targeted activation of striatal ChIs is able to directly drive DA release. To confirm recording site viability, we initially explored DA release in response to electrical stimuli (single pulses, 1p, or trains of 4 pulses, 4p at 25 Hz); evoked striatal DA release in rat did not vary with stimulation protocol in drug-free control conditions (Fig. 1*C*,*D*), consistent with previous observations in mice and guinea pig (Rice and Cragg, 2004; Zhang et al., 2009; Exley et al., 2012). DA release was strongly regulated by nAChRs: when nAChRs were inhibited by antagonist DHβE (1 μm), peak [DA]_o_ evoked by 1p was reduced and by 4p (25 Hz) was slightly facilitated (Fig. 1*C*,*D*), consistent with previous findings (Rice and Cragg, 2004; Zhang and Sulzer, 2004; Zhang et al., 2009). Thus, inhibition of nAChRs promoted the sensitivity of DA release to axonal activity (Fig. 1*D*; 2-way ANOVA drug × pulse number interaction *F*_1,11_ = 8.5, *p* = 0.014^a^; *post hoc* test: 1p versus 4p DHβE, *p* < 0.05). In the same recording sites, in the absence of DHβE, targeted activation of ChR2-expressing ChIs using blue light pulses (1p or 4p, 25 Hz) also evoked DA release and was insensitive to pulse number (Fig. 1*E–G*), as shown previously in mice using AAV5-packaged ChR2 (Threlfell et al., 2012). Light-activated DA release was abolished in the presence of DHβE (1 μm), showing the dependence on nAChRs. Since ChIs but not brainstem neurons express ChR2 in these animals (Fig. 1*A*), these data show that that targeted activation of ChIs alone can reproduce in rats the previously documented effects in mice of activating all striatal ACh systems on driving DA release (Fig. 1*E*,*F*; *n* = 4 slices from 3 rats). Evoked [DA]_o_ ranged from 0.2 to 1.2 μm for single electrical pulses and from 0.13 to 1.7 μm for single light pulses.

### Striatal ACh from brainstem afferents does not drive DA release

**Figure 2.**
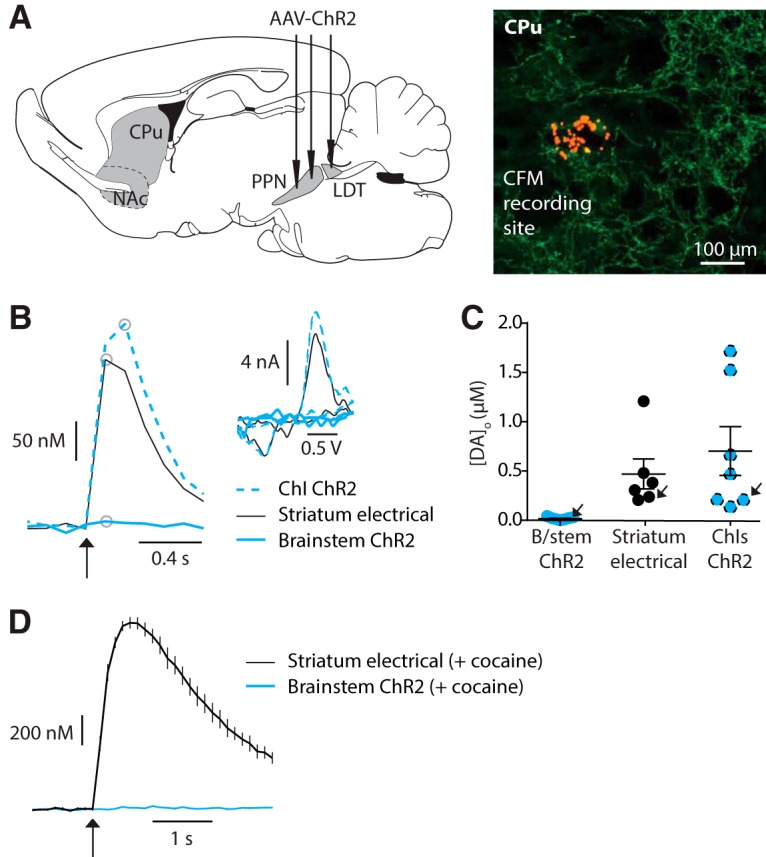
Light activation of striatal cholinergic brainstem afferents with brief stimuli does not reveal dopamine release. ***A***, Left, cartoon indicating PPN/LDT injections sites, and right, recording site labeled with red FluoSpheres in an area of cholinergic brainstem innervation indicated by ChR2-eYFP fluorescence. ***B***, Example profiles of [DA]_o_ (μm) versus time evoked by either light activation of ChIs (ChI ChR2, blue dashed, 1p), electrical stimulation of striatum (black solid line, 1p), or light activation of brainstem cholinergic afferents (brainstem ChR2, blue solid line, 10p 10 Hz). Inset, corresponding voltammograms for DA following each activation type for site indicated in ***A***, at time point indicated on profiles by gray circles. ***C***, Peak evoked [DA]_o_ (μm) for each recording site and stimulation methods, with mean ± SEM indicated. Arrows indicate data points and sites shown in ***B***. ***D***, Mean profiles of current detected at DA oxidation potential (± SEM) versus time evoked by striatal electrical stimulation (1p; black) or light stimulation of ChR2-expressing cholinergic brainstem afferents (10p 10 Hz; blue) in the presence of cocaine (5 μm; *n* = 10 observations from 3 sites).

To explore whether striatal ACh afferents from PPN/LDT can drive DA release in response to these brief stimulations, when activation of either ChIs alone (see Fig. 1) or all striatal ACh networks strongly drive DA release (Cachope et al., 2012; Threlfell et al., 2012), we injected an AAV2 containing ChR2-eYFP constructs into the right PPN/LDT of ChAT-cre rats (Fig. 2*A*). This protocol leads to ChR2-eYFP expression in ∼70% of PPN cholinergic neurons (Dautan et al., 2016). In contrast to the high density of ChR2-eYFP–expressing striatal fibers seen after striatal injections, striatal ChR2-eYFP expression after PPN/LDT injections was not visible by fluorescence imaging during recordings in live slices. Expression of ChR2 was corroborated only after recording, following injection of red FluoSpheres to mark recording sites using a published protocol (Brimblecombe and Cragg, 2015), followed by fixation, to confirm whether recordings were in an area of innervation by ChR2-expressing brainstem afferents (Fig. 2*A*).

In striatal sites with confirmed ChR2-eYFP–expressing brainstem fibers, brief light pulses did not evoke detectable levels of [DA]_o_ even with trains of 10 pulses (10 Hz; Fig. 2*B*,*C*). By contrast, electrical stimulation at the same recording sites reliably evoked DA release, proving site viability (Fig. 2*B*,*C*). Furthermore, light activation of the opposite hemisphere where ChIs were transfected with ChR2-eYFP also reliably evoked DA release, even with single pulses (Fig. 2*B*,*C*). To test if activation of cholinergic brainstem afferents was driving DA release below our DA detection threshold, we applied an inhibitor of DA uptake, cocaine (5 µM), to enhance any evoked DA transients. In the presence of cocaine, [DA]_o_ evoked by electrical stimuli (single pulses) were large and prolonged, but by contrast, light activation of brainstem afferents did not evoke detectable [DA]_o_ (Fig. 2*D*). These observations suggest that activation of cholinergic brainstem afferents alone does not generate a sufficient ACh source with these protocols in slices, to explain how striatal DA release is locally driven by striatal ACh.

### Brainstem ACh afferents do not modulate electrically evoked DA release

We tested the possibility that brainstem afferents might nonetheless be able to modulate presynaptic excitability of DA axons but in a manner that is below the threshold for directly driving DA release. We tested whether we could expose a role for brainstem ACh in the modulation of DA release evoked by other stimuli. We established a stimulus protocol in which DA release was driven by local electrical stimulation with or without a prior light pulse train in ChR2-expressing striatum. We hypothesized that if prior light stimulation caused ACh release to reach nAChRs on DA axons, then it might modulate nAChR activity and/or promote desensitization, and consequently change the frequency sensitivity of electrically evoked DA release that is critically determined by nAChRs (Cragg, 2006). We used a light pulse train (10p/10 Hz) that commenced 0.5 s before electrical stimulation to detect any effects of light stimulation alone, as well as subsequent effects on combined electrical stimulation.

**Figure 3.**
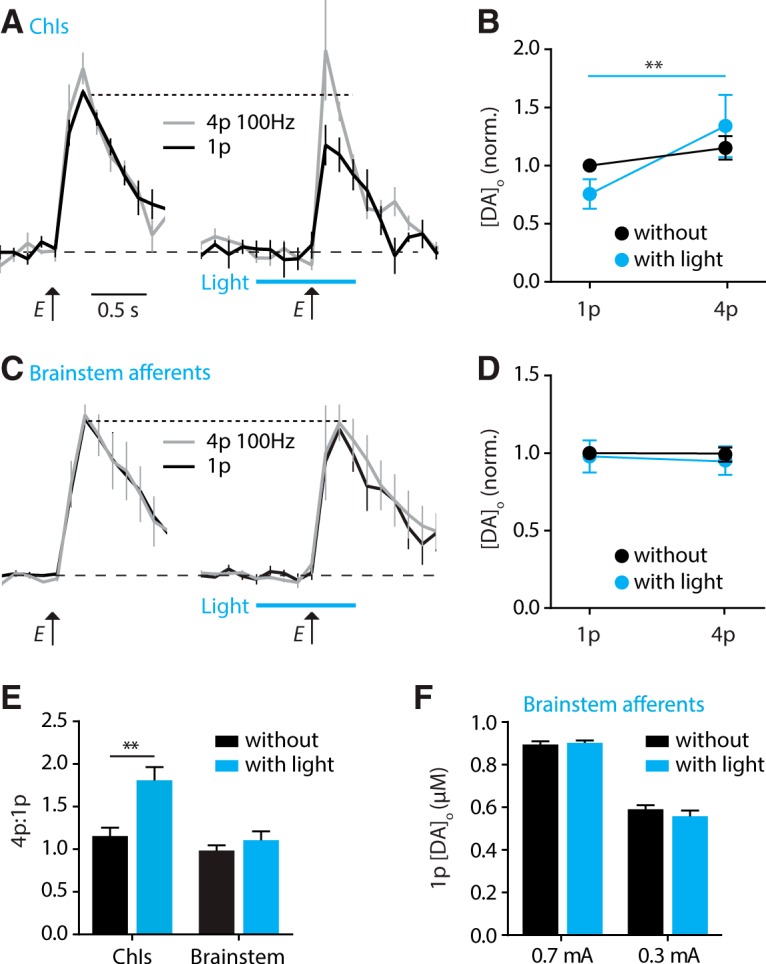
Light activation of striatal cholinergic interneurons but not brainstem afferents readily modulates dopamine release. ***A***, ***C***, Mean profiles of [DA]_o_ ± SEM versus time evoked by 1 or 4 pulses (100 Hz) of electrical (E) stimulation alone (left), or with stimulation with light (10p/10 Hz beginning 0.5 s before electrical; right) to stimulate ChR2 expressed in either ChIs (***A***) or brainstem afferents (***C***). Data are normalized to 1p control (without light). ***B***, ***D***, Summary data showing mean peak [DA]_o_ ± SEM evoked by the electrical stimulation normalized to control 1p, for ChR2-expressing ChIs (***B***) or brainstem afferents (***D***) with and without light activation (blue versus black). Two-way ANOVA with repeated measures with Sidak posttest comparisons of 1p versus 4p: **p < 0.01; N.S., *p* > 0.05. ***E***, Ratio of [DA]_o_ evoked by 4 versus 1 electrical pulses (100 Hz) with and without light stimulation (blue versus black) of either ChIs or striatal brainstem afferents. Light pre-stimulation of ChIs but not brainstem afferents significantly potentiated 4p:1p ratio of electrically evoked DA release (2-way ANOVA; Sidak posttest for with versus without light: ChI: *t*_18_ = 4.1, **p < 0.01; brainstem: *t*_18_ = 0.58, *p* > 0.05). ***F***, Mean peak 1p [DA]_o_ ± SEM, evoked by 0.7 mA and 0.3 mA electrical stimuli, with and without light prestimulation of brainstem afferents. Two-way ANOVA; Sidak posttest with versus without light: 0.7 mA: t_12_ = 0.29, *p* > 0.05; 0.3 mA: *t*_12_ = 1.2, *p* > 0.05. Data are in μm, *n* = 4 rats, *n* = 6 sites.

We first established the effects of combined light and electrical stimulation on striatal DA when ChR2 was expressed by ChIs. We selected striatal regions where the density of ChR2-eYFP–expressing fibers from ChIs was sparse, to be more comparable to the innervation by brainstem afferents. In regions sparsely innervated by ChR2-expressing ChIs, electrical stimuli alone readily evoked DA release, but trains of light pulses (10p/10 Hz) did not drive [DA]_o_ to a detectable level (see Fig. 3*A*). A prestimulation of ChIs with light pulses that commenced 0.5 s before an electrical stimulus did not evoke detectable [DA]_o_ but nonetheless modified [DA]_o_ evoked by electrical stimulation: there was a reduction in [DA]_o_ evoked by a single electrical pulse and a tendency toward a facilitation of [DA]_o_ evoked by a burst of electrical pulses (4p/100 Hz; compared to electrical stimulus alone; Fig. 3*A*,*B*; 2-way repeated measures ANOVA: light condition × pulse number interaction: *F*_1,6_ = 17.1, *p* = 0.006^b^). There was a resulting increase in the ratio of [DA]_o_ evoked electrically by 4p versus 1p (Fig. 3*A*,*B*; *post hoc* test, 1p versus 4p with light: *p* < 0.05). This modulation of evoked [DA]_o_ is consistent with reduced activation of nAChRs (Rice and Cragg, 2004; Zhang and Sulzer, 2004) at the time of electrical stimulation, confirming that light activation of ChIs that was subthreshold for directly driving DA release was nonetheless able to modulate nAChRs and DA release.

We then tested the effect of prior light stimulation of cholinergic brainstem afferents on subsequent electrically evoked DA release. Light activation of brainstem afferents in striatum did not drive detectable [DA]_o_ (as in Fig. 2), and furthermore, did not modulate [DA]_o_ evoked by electrical stimulation given 0.5 s later (Fig. 3*C*,*D*; 2-way repeated measures ANOVA, effect of light: *F*_1,4_ = 0.19, *p* = 0.68; pre-flash × pulse number interaction, *F*_1,4_ = 0.02, *p* = 0.88^c^). The ratio of [DA]_o_ evoked electrically by 4p versus 1p was not enhanced by light prestimulation of brainstem afferents (Fig. 3*E*, 2-way ANOVA, effect of region: *F*_1,18_ = 8.7, *p* = 0.004; posttest, with versus without pre-pulse: ChIs, *p* < 0.01^d^).

Finally, in the event that subtle effects of prior ACh action at nAChRs were masked by the stronger subsequent electrical stimuli, we tested whether we could expose an effect of a light prestimulation for a lower electrical stimulation current. A lower electrical current evoked [DA]_o_ that were ∼two-thirds of those previously, but nonetheless, prestimulation of ChR2-expressing brainstem afferents with light pulse trains did not modify [DA]_o_ further (Fig. 3*F*, 2-way ANOVA: effect of light: *F*_1,12_ = 0.2, *p* = 0.67^e^).

## Discussion

We show here, using specific targeting of ChR2 to either striatal ChIs or brainstem cholinergic neurons of PPN/LDT, that the powerful effects of striatal ACh and nAChRs on DA release reported previously *ex vivo* can be reproduced by targeted activation of ChIs. Striatal ChIs and nAChRs can directly drive DA release, and also modulate DA release driven by activity generated in DA neurons, shown here in rats, as reported previously in mice using nonspecific ACh activation (Rice and Cragg, 2004; Zhang and Sulzer, 2004; Cachope et al., 2012; Threlfell et al., 2012). By contrast, while PPN/LDT nuclei can modulate somatodendritic DA neuron activity (Dautan et al., 2016), we could not find any evidence that similar stimulation protocols could recruit PPN/LDT afferents in striatum to regulate DA release from axons.

### Two cholinergic sources for regulation of striatal DA

ACh is well known to modulate DA transmission through two major cholinergic pathways that have previously been assumed to be distinct. First, roles of the cholinergic brainstem for modulating DA neuron activity and consequent DA release have previously been established. Cholinergic neurons of the brainstem make synaptic contacts with midbrain DA neurons (Beninato and Spencer, 1988; Bolam et al., 1991), and direct activation of midbrain ACh receptors or PPN/LDT cholinergic axons modulates the discharge properties of DA neurons and, consequently, the downstream release of DA in the striatum (Blaha and Winn, 1993; Dautan et al., 2016). These studies indicate that cholinergic PPN/LDT neurons are able to modulate DA release via inputs to midbrain DA neurons. Second, a role for striatal acetylcholine systems, nAChRs, and nicotine in modulating DA axonal release directly has become well documented, and striatal ACh has been shown to bypass midbrain DA neurons and directly drive DA release (Rice and Cragg, 2004; Zhang and Sulzer, 2004; Cragg, 2006; Cachope et al., 2012; Threlfell et al., 2012). The neurons responsible for local striatal modulation were assumed to be only the intrinsic interneurons, the ChIs. However, the identification of cholinergic afferents in striatum arising from the PPN/LDT (Dautan et al., 2014) suggested that brainstem inputs might be partly responsible for local striatal modulation of DA by contributing as an additional source of striatal ACh. However, we were unable to obtain any evidence here for either ACh release or DA modulation by presynaptic nAChRs following PPN/LDT stimulation.

### Cholinergic interneurons but not brainstem afferents account for rapid regulation of striatal DA by nAChRs

Our experiments explored whether protocols that have been used previously to activate striatal ACh systems, and that drive and powerfully modulate striatal DA via nAChRs, primarily involve ChIs or brainstem afferents. Our data indicate that whereas ACh from ChIs could account for previously documented effects of nAChRs on striatal DA, we could not show the same was true for brainstem afferents. Single light flashes in ChIs are sufficient to drive DA release directly, but by contrast, single flashes or even more sustained stimulation of brainstem afferents did not allow us to detect any DA. We were unable to find any further signs of modulation of nAChRs on DA axons in a protocol designed to explore whether activation of brainstem afferents is capable of modulating DA release evoked by other stimuli. As a positive control, we showed that pre-activation of sparsely labeled ChIs, that in isolation was unable to drive DA release, was nonetheless able to modulate electrically evoked DA release, and significantly increase the 4p:1p ratio. This increase in 4p:1p ratio is consistent with a decrease in activation of nAChRs on DA axons at the point of electrical stimulation. This decrease in nAChR activity could result from initial ACh release leading to a depression in ACh re-release by the subsequent electrical pulse, or to some nAChR desensitization, to which nAChRs are particularly susceptible (Zhou et al., 2001; Quick and Lester, 2002; Rice and Cragg, 2004). By contrast, prestimulation of brainstem afferents with light had no measurable effect on subsequent electrically evoked [DA]_o_ or on 4p:1p ratio.

These data suggest that any ACh released by activation of brainstem afferents alone is unable to reach sufficient levels to modulate nAChRs on DA axons. The more readily detectable role for ChIs in gating striatal DA, and also for cortical or thalamic glutamate inputs to ChIs (Kosillo et al., 2016) than for brainstem inputs, is in keeping with the relative visibility of striatal YFP expression noted in 300-µm-thick slices under the recording microscope. This conclusion is also consistent with the finding that deletion of brainstem cholinergic neurons does not affect electrically evoked dopamine release in striatal slices (Patel et al., 2012). We cannot exclude the possibility that ACh might not be released from PPN/LDT terminals in striatum in *ex vivo* slices. This caveat does not detract from the principal conclusion that striatal ChIs are entirely sufficient to account for current observations of the modulation of DA by striatal ACh. Our findings do not necessarily preclude brainstem afferents from locally regulating DA release during other conditions, e.g., *in vivo*, when they might modulate the dynamic activity *in vivo* of networks of striatal neurons or ChIs (Dautan et al., 2018), which might in turn subsequently regulate DA release.

### Summary and conclusions

In summary, we show that ChIs readily drive DA release in rat, as shown previously in mouse, and that striatal modulation of DA transmission by nAChRs and nicotine can readily be reproduced by targeted activation of ChIs but not brainstem afferents. Brainstem afferents were not sufficient to drive or modulate striatal DA release under the experimental paradigms used here as previously, in which ChI function dominates.

## Synthesis

Reviewing Editor: Lorna Role, Stony Brook Decisions are customarily a result of the Reviewing Editor and the peer reviewers coming together and discussing their recommendations until a consensus is reached. When revisions are invited, a fact-based synthesis statement explaining their decision and outlining what is needed to prepare a revision will be listed below. The following reviewer(s) agreed to reveal their identity: Thomas Gould, Frances Leslie.

Synthesis:

In the manuscript entitled, “Cholinergic brainstem afferents do not account for modulation of dopamine by striatal nicotinic receptors and cholinergic interneurons,” the authors present evidence (through optogenetic methods in the rat brain) that stimulation of cholinergic striatal interneurons (ChIs) --but not cholinergic brainstem inputs from the PPN/LDT -- are sufficient to mediate the effects of cholinergic modulation of striatal dopaminergic (DA) transmission. The data are generally robust, clearly presented, and quite compelling. The challenges of proving a negative finding (i.e. that ChP PN/LDT inputs do not affect DA transmission) are reasonably well addressed.

The reviewers agreed that there are a few additional experimental controls that should be included to seal minor gaps in the argument. These additional controls would greatly contribute to the significance of the manuscript. There are 2 sets of Major issues, both related to additional controls.

Major:

1. Provide additional, direct evidence for the differential labeling by AAV2 vs AAV5 as this is critical to the main point of the paper that ChIs regulate striatal DA but Ch PPT/LKDT inputs do not have a significant effect. Specifically, we suggest that the authors include a few images of AAV-positive and AAV-negative cholinergic neurons in PPN/LDT after injections of AAV5 and AAV2, respectively, in the revised manuscript as this would greatly enhance the quality of the manuscript.

It seems reasonable to assume based on other studies that AAV2, but not AAV5, injections into striatum only target the ChIs themselves in ChAT-Cre rats and do not also label other cholinergic terminals such as those from the cholinergic neurons of PPN/LDT. However, given the critical importance of this assumption, the authors should provide more direct evidence for this.

In line 186, the authors state that some of this evidence is to be found in Dautan et al. (2016). Is this from the experiments in Supplemental Figure 2 in that paper, in which it was stated that after injections of AAV2 into striatum, “no retrograde transduction of cholinergic neurons was detected?”

Do the authors know if this was done by failing to find fluorescence in ChAT-immunopositive cells of PPN/LDT, after dorsal striatal injections of AAV2-eYFP or AAV-ChR2-eYFP?

Similarly, do the authors have primary data to support the assertion that striatally injected AAV5 infects cholinergic neurons in PPN/LDT as well as the ChIs (as implied in line 83).

2. Provide specific criteria for ‘sufficient’ labeling of brainstem terminals to guarantee that there was effective activation of these terminals. We suggest additional controls to provide support for the functional activation of PPN/LDT terminals.

Given that the tegmental terminals are much sparser than those of the interneurons, it is more difficult to interpret a negative finding. The authors understand this and have taken measures to ensure that only data from recordings in the regions of tegmental terminal projections were included in the data. However, no criteria were provided as to how such regions are defined. Please provide specific criteria.

It would be best if the authors provided validation for the transduction/optogenetic activation of PPN/LDT afferent axons/terminals after direct brainstem injection by some technique other than simply fluorescent arborization in the striatum.

Perhaps the authors could demonstrate an effect of this manipulation on the a.) modulation of DA neuronal firing activity in VTA (Dautan et al., 2016; others) or in SNc (Futami et al., Neurosci Res., 21 (1995), pp. 331-342); b.) modulation of DA release in NAc (Floresco et al., Nat. Neurosci, 6 (2003), pp. 968-973); c.) function of any cell type in the striatum.

Without such control data it is uncertain whether the transduction and/or optogenetic manipulation of cholinergic neurons in the PPN/LDT resulted in the activation and release of ACh from their terminals in the striatum, and therefore whether the negative results of this manipulation reflects a lack of effect on striatal DA release.

Other studies have shown that optogenetic activation of distal axons/synaptic terminals in the striatum is sufficient to regulate DA release (Kosillio et al., Cereb Cortex 2016 26(11): 4160-4169), perhaps it is not for the terminals of these brainstem afferents. In this vein, the observation that YFP was not visible in 300 micron-thick sections could be discussed.

Additional Minor points that should be addressed by changes to text and/or explanation by the authors:

Figure 1 seems to be very similar to previously published data - both the electrical (Rice and Cragg, Nature Neuroscience 2004 7:583-584) and optical (Threlfell et al., Neuron 2012, 75: 58-64) activation of the striatum / ChIs has been reported, and while it is useful to establish the difference between the positive effects of ChI stimulation vs. those of brainstem stimulation, I wonder if they could be subsumed in Figure 2.

113 - “Light activation of these inputs can successfully drive a cholinergic output from terminals.” Is this previous data or new? This could help address one of the major points above.

159 - Since the 300 micron-thick striatum sections didn't show YFP fluorescence after AAV-ChR2-eYFP into PPN/LDT, was the signal visible in the 40-micron thick sections simply because they were thinner or because they were immunoreacted with anti-GFP antibodies?

163 - In the methods for this manuscript, it mentions that the PPN/LDT afferents target the central to median striatum - is this the same region as the ‘dorsal striatum,’ described as the striatal injection source used in part to determine the absence of retrograde transduction of PPN/LDT neurons in Gautan et al., 2016?

165 - LDT not LTD

183 - add ‘similar to’ in front of ‘previous observations’

-- the statistical analysis table indicates that the comparison for analysis b is with and without DhβE, which was not used in this experiment.

## Author Response

Reply to Reviewers

General comments: We are glad to see the general enthusiasm for our work from the reviewers. We have paid careful attention to the two main points raised for discussion that seal the minor gaps in the work as presented, and have detailed our responses below in blue.

1. “Provide additional, direct evidence for the differential labeling by AAV2 vs AAV5 as this is critical to the main point of the paper that ChIs regulate striatal DA but Ch PPT/LKDT inputs do not have a significant effect. Specifically, we suggest that the authors include a few images of AAV-positive and AAV-negative cholinergic neurons in PPN/LDT after injections of AAV5 and AAV2, respectively, in the revised manuscript as this would greatly enhance the quality of the manuscript.”

RESPONSE: In this study, we conducted injections of only AAV2, and not AAV5. AAV5 was used previously in other papers in mice (Threlfell et al 2012; Cachope et al 2012). We used AAV2 here to drive only anterograde expression, to generate ChR2 expression specifically in either striatal ChIs after striatal injection, or in PPN/LDT and their projections after PPN/LDT injection. We used previously published methods (Dautan et al 2016), in which it was identified that striatal injection of AAV2-eYFP leads to expression restricted to ChIs and does not result in retrograde expression in PPN/LDT. AAV2 is transported in anterograde but not retrograde manner (Salegio et al 2013 PMID 22418061). We have added a citation to this manuscript to further support this premise. We have also corroborated that we do not detect retrograde expression of ChR2-eYFP in PPN/LDT after striatal AAV2-ChR2-eYFP injections (see new Figure panel 1A).

“It seems reasonable to assume based on other studies that AAV2, but not AAV5, injections into striatum only target the ChIs themselves in ChAT-Cre rats and do not also label other cholinergic terminals such as those from the cholinergic neurons of PPN/LDT. However, given the critical importance of this assumption, the authors should provide more direct evidence for this.”

RESPONSE: If striatal AAV2 injections were to drive striatal expression of ChR2 in cholinergic terminals of PPN/LDT, we would see expression in cell bodies in PPN/LDT which we do not. As stated above, we have revised Figure 1 to include new images to show this here as previously (Dautan et al 2016).

“In line 186, the authors state that some of this evidence is to be found in Dautan et al. (2016). Is this from the experiments in Supplemental Figure 2 in that paper, in which it was stated that after injections of AAV2 into striatum, ‘no retrograde transduction of cholinergic neurons was detected?’ Do the authors know if this was done by failing to find fluorescence in ChAT-immunopositive cells of PPN/LDT, after dorsal striatal injections of AAV2-eYFP or AAV-ChR2-eYFP?

RESPONSE: This is correct. In that manuscript, no retrograde transduction of cholinergic neurons was detected after striatal injections of AAV2-EF1a-DIO-YFP by Dautan et al 2016. However, we have now confirmed that this is also the case here using AAV2-EF1a DIO-ChR2-eYFP and clarified this by adding new panels to Figure 1, and cited these findings at the opening of the Results on P8.

Similarly, do the authors have primary data to support the assertion that striatally injected AAV5 infects cholinergic neurons in PPN/LDT as well as the ChIs (as implied in line 83).

RESPONSE: We did not inject AAV5 into ChAT-Cre rats in this study, but AAV5 is well known and used for it ability to transduce expression both anterogradely and retrogradely.

2. Provide specific criteria for ‘sufficient’ labeling of brainstem terminals to guarantee that there was effective activation of these terminals. We suggest additional controls to provide support for the functional activation of PPN/LDT terminals:

Given that the tegmental terminals are much sparser than those of the interneurons, it is more difficult to interpret a negative finding. The authors understand this and have taken measures to ensure that only data from recordings in the regions of tegmental terminal projections were included in the data. However, no criteria were provided as to how such regions are defined. Please provide specific criteria.

RESPONSE: In brief, we sampled the striatum in multiple regions, blind with respect to brainstem innervation and labelled those sites with FluoSpheres. If any of those sites were then situated within a region that overlapped with YFP-expressing neuropil, detectable in thin and mounted re-sections, then data were included. We have clarified our description on P7.

It would be best if the authors provided validation for the transduction/optogenetic activation of PPN/LDT afferent axons/terminals after direct brainstem injection by some technique other than simply fluorescent arborization in the striatum. Perhaps the authors could demonstrate an effect of this manipulation on the a.) modulation of DA neuronal firing activity in VTA (Dautan et al., 2016; others) or in SNc (Futami et al., Neurosci Res., 21 (1995), pp. 331-342); b.) modulation of DA release in NAc (Floresco et al., Nat. Neurosci, 6 (2003), pp. 968-973); c.) function of any cell type in the striatum. Without such control data it is uncertain whether the transduction and/or optogenetic manipulation of cholinergic neurons in the PPN/LDT resulted in the activation and release of ACh from their terminals in the striatum, and therefore whether the negative results of this manipulation reflects a lack of effect on striatal DA release.

RESPONSE: We want to make two separate points (a) and (b).

(a) A central aim of the manuscript was to evaluate whether ChIs or PPN/LDT inputs could account for the key effects of striatal ACh on DA release which have been previously been documented and that have changed our understanding of the scope of actions of ACh on DA (Threlfell et al 2012; Cachope et al 2012). In previous studies, the use of AAV5 will have transduced all striatal ACh systems. Here we set out to test whether under stimulation conditions used previously (Threlfell et al 2012), the regulation of DA can be accounted for by activation of ChIs only, and/or by actions of PPN/LDT inputs. We show firstly that light activation of ChIs alone can reproduce the previously documented effects of activating all striatal ACh systems on DA release. We then show that under these conditions, there is no similar effect of light flashes to ChR2-expressing PPN/LDT terminals. In other words, the actions of ChIs alone are sufficient to account for the control of DA by ACh. This principal conclusion holds true, regardless of whether or not PPN/LDT systems are being sufficiently activated to drive ACh release. Our manuscript intended to make this point, rather than testing whether PPN/LDT inputs are being effectively activated or not by the stimuli that are known to modulate DA. We have now clarified this intended emphasis throughout the manuscript, e.g. in Abstract, emphasis of Results on P8, in Discussion P12 end, start P13,P14.

(b) With regards to whether PPN/LDT inputs are being activated in slices under these conditions:- Our conclusion that ChIs are sufficient to account for the documented control of DA by ACh, holds true regardless of whether or not ACh can be released from PPN/LDT afferent in striatal slices. Whether or not ACh is being released from PPN/LDT terminals is not then critical to our findings, but we appreciate that some discussion of this issue is warranted. Thus we conducted a series of pilot experiments to explore whether we can find evidence for ACh release from brainstem afferents in striatal slices, hence the delay in our responding. As of yet, we have been unable to establish whether we can drive a detectable release of ACh from these terminals in striatal slices. As a result, we have now added sentences to the manuscript on P14, to state that “We cannot exclude the possibility that ACh might not be released from PPN/LDT terminals in striatum in ex vivo slices. This caveat does not detract from the principal conclusion that striatal ChIs are entirely sufficient to account for current observations of the modulation of DA by striatal ACh.”.

Other studies have shown that optogenetic activation of distal axons/synaptic terminals in the striatum is sufficient to regulate DA release (Kosillio et al., Cereb Cortex 2016 26(11): 4160-4169), perhaps it is not for the terminals of these brainstem afferents. In this vein, the observation that YFP was not visible in 300 micron-thick sections could be discussed.

RESPONSE: We thank the reviewer for this suggestion, and agree it is interesting that glutamate inputs from cortex and thalamus can regulate DA (via cholinergic interneurons, Kosillo et al 2016) whereas those from brainstem do not. YFP expression was certainly visible in 300 micron sections in those experiments. We have added a sentence of discussion of this point on P13, “The more readily detectable role for ChIs in gating striatal DA, and also for cortical or thalamic glutamate inputs to ChIs (Kosillo et al., 2016) than for brainstem inputs, is in keeping with the relative visibility of striatal YFP expression noted in 300 µm-thick slices under the recording microscope.”

Additional Minor points that should be addressed by changes to text and/or explanation by the authors:

Figure 1 seems to be very similar to previously published data - both the electrical (Rice and Cragg, Nature Neuroscience 2004 7:583-584) and optical (Threlfell et al., Neuron 2012, 75: 58-64) activation of the striatum / ChIs has been reported, and while it is useful to establish the difference between the positive effects of ChI stimulation vs. those of brainstem stimulation, I wonder if they could be subsumed in Figure 2.

RESPONSE: We have chosen to maintain separate figures, because previous work was conducted in mice and did not distinguish the source of ACh afferents. We want to make the distinct point that targeted activation of ChIs here for the first time can drive and modulate DA release, rather than subsume it into the next figure.

113 - “Light activation of these inputs can successfully drive a cholinergic output from terminals.” Is this previous data or new? This could help address one of the major points above.

RESPONSE: This should read “Light activation of these inputs can successfully drive a cholinergic output from terminals in VTA” and has been modified to do so.

159 - Since the 300 micron-thick striatum sections didn't show YFP fluorescence after AAV-ChR2-eYFP into PPN/LDT, was the signal visible in the 40-micron thick sections simply because they were thinner or because they were immunoreacted with anti-GFP antibodies?

RESPONSE: Fluorescence could be seen after fixation, re-sectioning and mounting, without additional antibodies. We have modified the description of imaging in the methods and at other places to make our processes clearer, including stating when we imaged ‘ChR2-eYFP fluorescence’ to ensure this clear in the manuscript.

163 - In the methods for this manuscript, it mentions that the PPN/LDT afferents target the central to median striatum - is this the same region as the ‘dorsal striatum,’ described as the striatal injection source used in part to determine the absence of retrograde transduction of PPN/LDT neurons in Gautan et al., 2016?

RESPONSE: These regions are overlapping. Moreover, we reassessed here with our injections in this study, and corroborated the absence of retrograde transduction after striatal injections to regions recorded after striatal injections.

165 - LDT not LTD

RESPONSE: Thanks -- corrected

183 - add ‘similar to’ in front of ‘previous observations’

RESPONSE: This sentence has now been modified to place greater emphasis on testing whether targeted activation of ChIs is sufficient to modify DA release.

-- the statistical analysis table indicates that the comparison for analysis b is with and without DhβE, which was not used in this experiment.

RESPONSE: Thanks for spotting this error. Corrected.
